# Supplementary material for: Atomic Layer Deposition of Antibacterial Nanocoatings: A Review
Source: Antibiotics (Basel). 2023 Nov 24;12(12):1656. doi: 10.3390/antibiotics12121656 (PMC10740478; doi:10.3390/antibiotics12121656)
Supplement: Supplementary file 1 [file antibiotics-12-01656-s001.zip › antibiotics-2697842-supplementary.pdf]

Atomic layer deposition of antibacterial nanocoatings: a review

Denis Nazarov [1,2,*], Lada Kozlova [2], Elizaveta Rogacheva [3], Ludmila Kraeva [3], and Maxim Maximov [1]

[1] Peter the Great Saint Petersburg Polytechnic University, Polytechnicheskaya, 29, 195221, Saint Petersburg, Russian Federation; dennazar1@yandex.ru (D.N.), maximspbstu@mail.ru (M.M.)
[2] Saint Petersburg State University, Universitetskaya nab, 7/9, 199034, Saint Petersburg, Russian Federation; lada.kozlova.20@mail.ru (L.Ko.)
[3] Saint-Petersburg Pasteur Institute of Epidemiology and Microbiology, 14 Mira Street, 197101, Saint Petersburg, Russia; lykraeva@yandex.ru (L.Kr.), elizvla@yandex.ru (E.R.)
* Correspondence: dennazar1@yandex.ru; Tel.: +7-812-428-4033

1. Methods

1.1. Literature Search

The first comprehensive electronic search was carried out using the Scopus database on 18 August 2023. In addition, a global screening was performed using Google Scholar and ALD websites. Articles were searched using combinations of the following keywords: atomic layer deposition and antibacterial or bactericidal. Searches were restricted to articles and communications published in English. The last search date was 5 September 2023. The search and analysis of the data was partly done using PRISMA (Preferred Reporting Items for Systematic Review and Meta-Analyses).

1.2. Inclusion and Exclusion Criteria

The inclusion criteria were—(1) ALD should be used to produce the entire coating or used as a separate production step; (2) Antibacterial test results must be contained; (3) the conference proceedings, reviews, books and book chapters were excluded.

1.3. Study Selection

Titles, abstracts and keywords were screened to assess the suitability of the search results. The full-text papers selected during screening were then downloaded and analysed to assess whether or not they met the inclusion criteria.

1.4. Data Extraction

Extracted information for initial analysis included — (1) The main purpose of the study; (2) The type of substrate; (3) The composition and thickness of the coatings; (4) ALD conditions (temperatures and precursors); (5) Type of bacteria and testing method; (6) The list of methods for studying samples; (7) The main results.

1.5. Analysis of the extracted data

The extracted data were summarised in tables and diagrams, and then analysed, including comparisons, systematisation, generalisation, and highlighting the most interesting and significant findings. On this basis, the text of this review was prepared.

1.6. Search Results

A total of 148 studies were identified using Scopus database (Figure S1). After screening the types of papers, 19 studies were excluded. After titles, abstracts and key words screening 73 papers were also excluded. As a result, 56 studies were selected for full-text analysis. 54 papers were accessible. After full-text analysis, only 46 articles remained, but 22 new relevant articles were identified based on related references and additional searches on websites and Google Scholar. Finally, results of 68 articles and communications were included in this review.

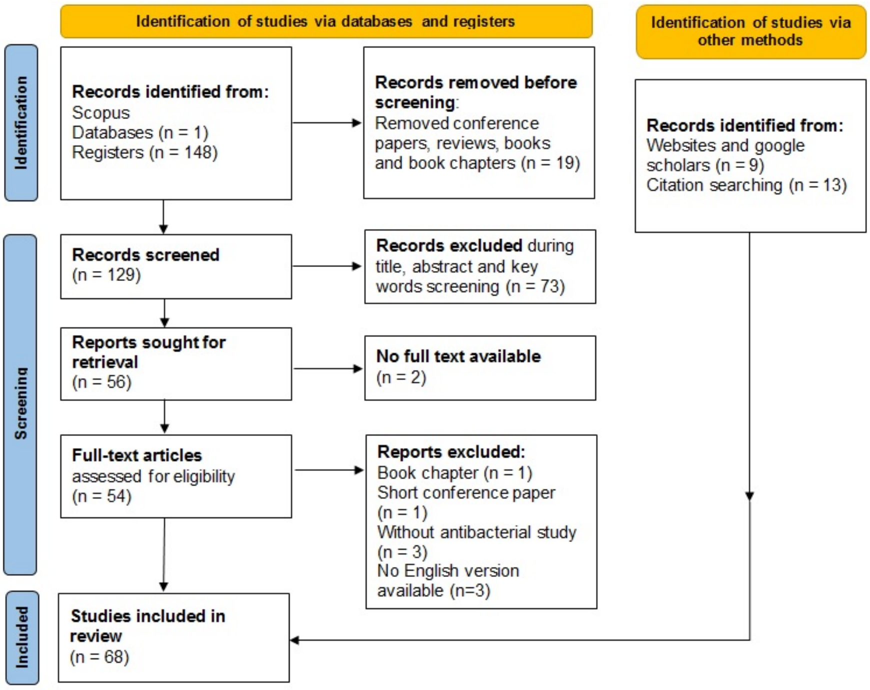

Figure S1. Flow diagram of the systematic literature search.
